# Supplementary figures and images for: Virus subtype-specific suppression of MAVS aggregation and activation by PB1-F2 protein of influenza A (H7N9) virus
Source: PLoS Pathog. 2020 Jun 8;16(6):e1008611. doi: 10.1371/journal.ppat.1008611 (PMC7302872; doi:10.1371/journal.ppat.1008611)

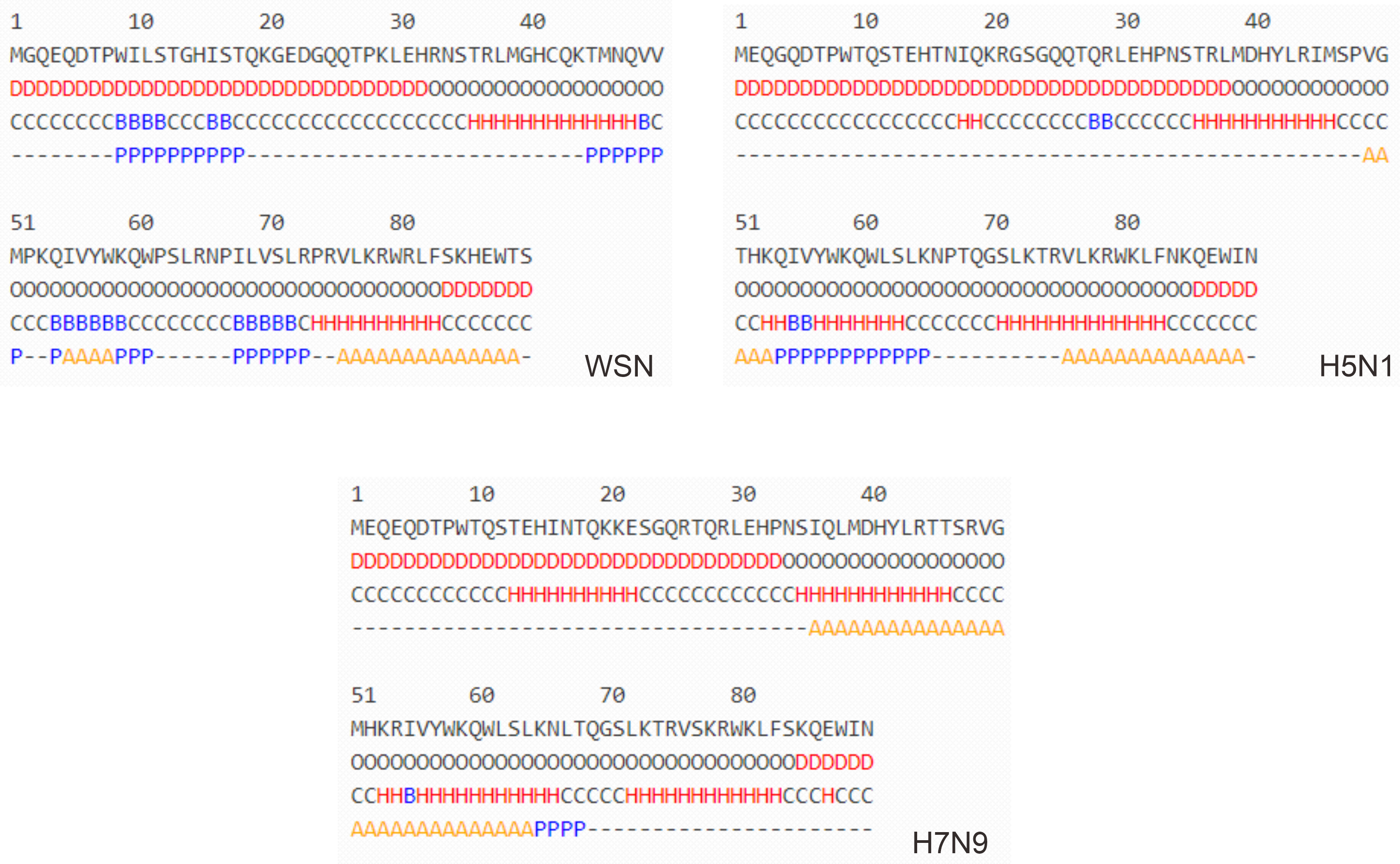

Supplement: S1 Fig — Amino acid sequences of WSN, H5N1 and H7N9 PB1-F2 were analyzed for putative cross β sheet structure using PASTA 2.0 (http://protein.bio.unipd.it/pasta2/). Predicted secondary structure and putative parallel or anti-parallel cross β-pairing were aligned to residues. P: parallel aggregation. A: anti-parallel aggregation. −: non-aggregating residue. D: disordered residue. O: ordered residue. H: α-helix residue. B: β-strand residue. C: coiled residue. (TIF) [file ppat.1008611.s001.tif]

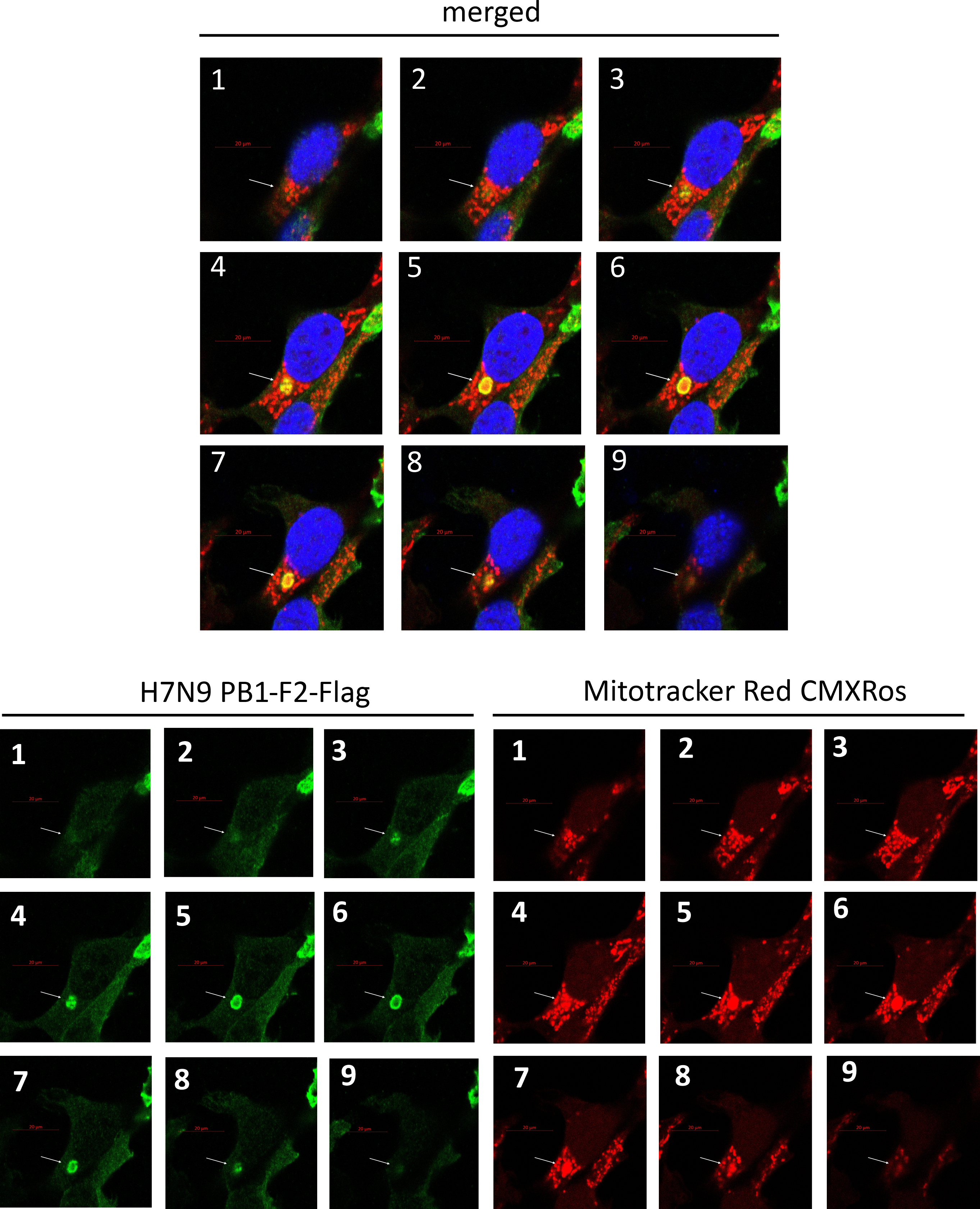

Supplement: S2 Fig — HEK293T cells were transfected with 1 μg of H7N9 PB1-F2-Flag expression construct for 48 hours followed by Mitotracker Red CMXRos staining, fixation and immunostaining with anti-Flag and DAPI as in Fig 3A. The stained cells were analyzed by confocal microscopy for Z-stack imaging. Images 1 to 9 represented nine images captured through Z-axis perpendicular to the image plane spaced by 0.7 μm. Arrows indicated distinct mitochondrial clusters co-localized with H7N9 PB1-F2. (TIF) [file ppat.1008611.s002.tif]

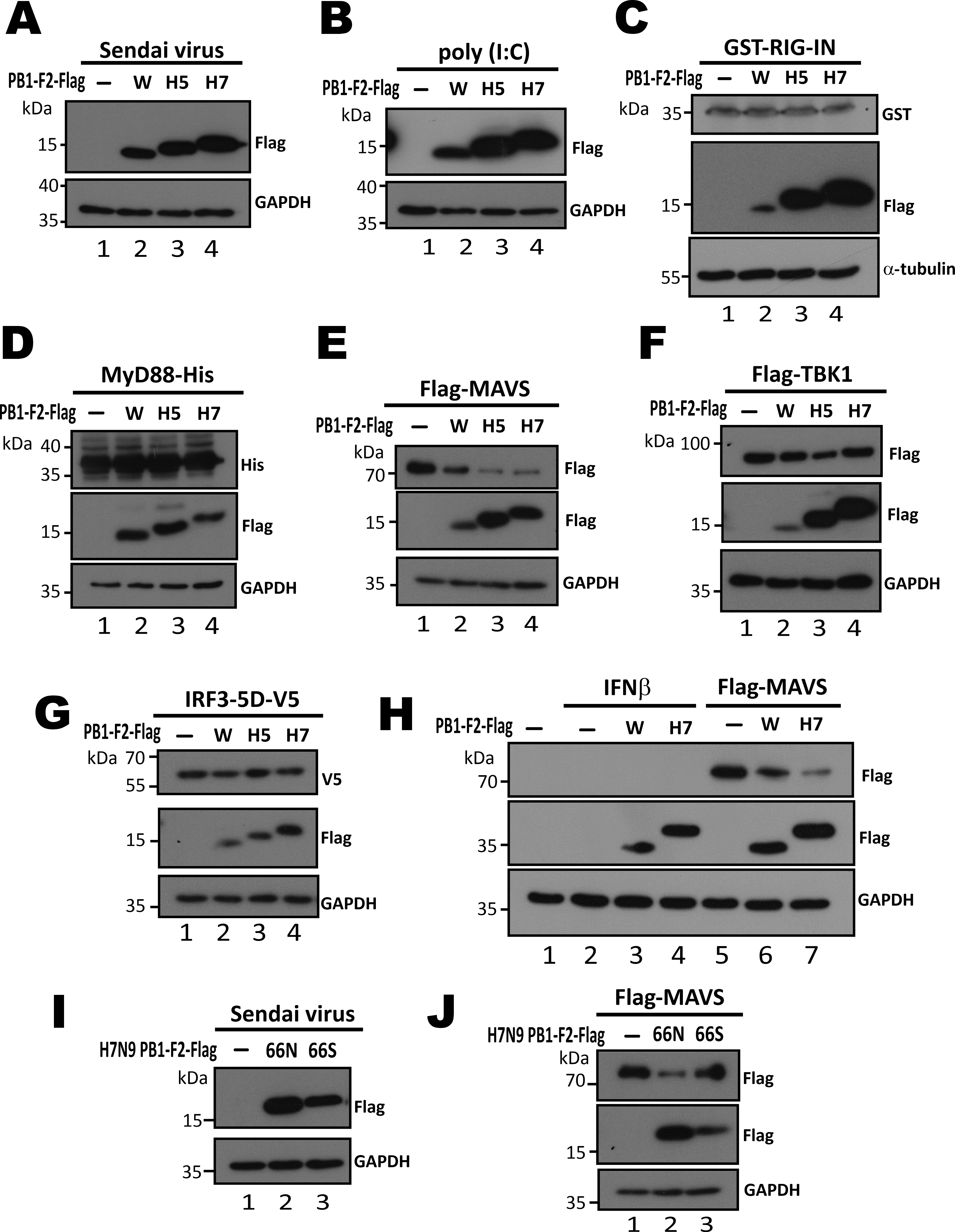

Supplement: S3 Fig — Samples of the same conditioned HEK293T cells as described in Fig 3 were subjected to total protein sample extraction followed by SDS-PAGE and Western blot analysis. (A) Protein samples of HEK293T cells transfected with PB1-F2-Flag plasmids and infected with Sendai virus as in Fig 3A were tested for expression level of PB1-F2-Flag normalized to GAPDH level. (B) Protein samples of HEK293T cells transfected with PB1-F2-Flag plasmid and poly (I:C) as in Fig 3B were tested as above. (C) HEK293T cells were transfected with PB1-F2-Flag and GST-RIG-IN plasmids as in Fig 3C. (D) Cells were transfected with MyD88-His plasmid as in Fig 3D. (E) Flag-MAVS was expressed as in Fig 3E, 3H and 3I. (F) Flag-TBK1 was expressed as in Fig 3F. (G) IRF3-5D-V5 was expressed as in Fig 3G. (H) Cells were treated with IFNβ or transfected with Flag-MAVS plasmid as in Fig 3J. (I) H7N9 PB1-F2 66N-Flag or H7N9 PB1-F2-66S-Flag was expressed. Cells were infected with Sendai virus as in Fig 3K. (J) Flag-MAVS was expressed as in Fig 3L. (TIF) [file ppat.1008611.s003.tif]

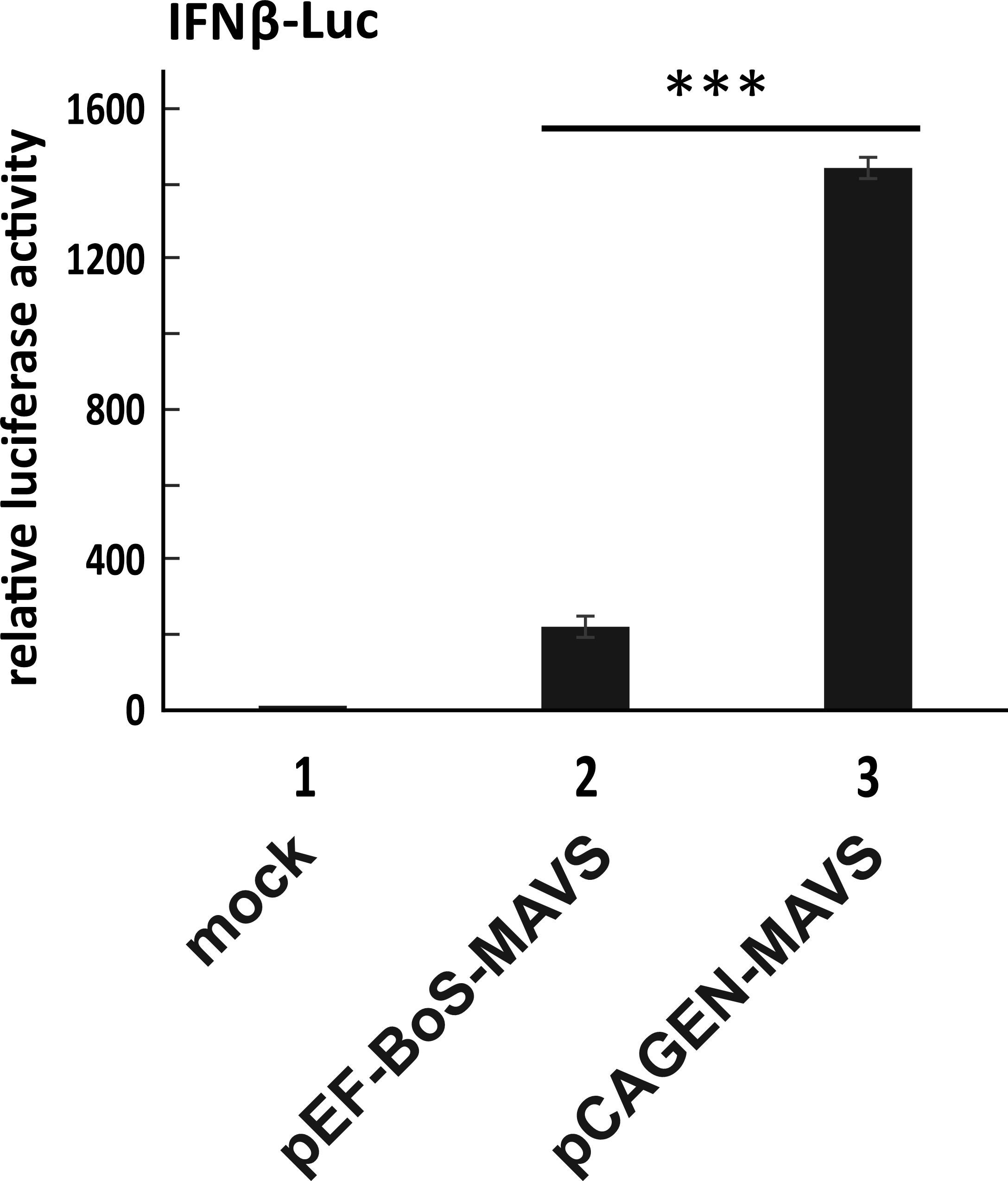

Supplement: S4 Fig — HEK293T cells in 24-well plates were transfected with 50 ng pCAGEN-myc-MAVS or pEF-Bos-Flag-MAVS plus 100 ng p125-Luc and 10 ng pRL-TK. After 48 hours, cells were harvested for dual-luciferase reporter assay. (TIF) [file ppat.1008611.s004.tif]
