# Supplementary material for: Virus subtype-specific suppression of MAVS aggregation and activation by PB1-F2 protein of influenza A (H7N9) virus
Source: PLoS Pathog. 2020 Jun 8;16(6):e1008611. doi: 10.1371/journal.ppat.1008611 (PMC7302872; doi:10.1371/journal.ppat.1008611)
Supplement: Attachment — Submitted filename: Response-PP-4May2020.docx [file ppat.1008611.s005.docx]

**Point-to-point response to reviewers’ comments** (PPATHOGENS-D-20-00243)

Reviewer #1: The study is largely based on over-expression experiments in 293T cells and a clear correlation between the protein amount of the three PB1-F2s and the respective phenotype investigated is obvious (e.g. Fig 6C and g, 7A/B). Interestingly, somewhat similar expression was achieved in Fig. 8C.

Luciferase reporter assays require transfection controls to assure comparable protein levels (e.g. Fig. 4a-l)

Response: Protein expression controls have been added to Fig. S3.

In several panels only H7 PB1-F2 is tested and the matching WSN PB1-F2 controls are missing. This needs to be included to point out the strain specific effects the authors are claiming (Fig 5, Fig. 10b d and e)

Response: We have repeated infection experiments in THP-1 cells with WSN WT and WSN ΔF. The new results are in Fig. 4D, 5D, 9C and 9F.

The quantification of Western blots in Fig. 7a does not appear to match with the actual bands

Response: This has now been fixed in the new Fig. 6A.

All experiments require indication of independent repeats, biological and technical replicates and statistical methods applied in the respective figure legends.

Response: The information requested has been added to the figure legends.

The quality of western blots needs to be improved (some blots are clearly to faint (Fig 6g aMAVS, 7a GAPDH and numerous are plain overexposed 7a flag, 6a GAPDH, 6c flag, 9BC and D V5). Often it is unclear if the signal is within the linear range. Additionally it remains elusive if the absolute amounts of PB1-F2 and MAVS are comparable to infection/cellular expression conditions.

Response: Better blots have been provided for Fig. 5A, 5F, 5G, 6A and 8. We tried to make sure the expression levels are as close as possible to those in infection or cell-endogenous expression.

How was aggregation of MAVS distinguished from overexpression in Fig 8C?

Response: MAVS overexpression can result in aggregation of MAVS and in turn activation of type I IFN. MAVS aggregation looks round and concentrated as demonstrated by another group (Methods Mol Biol 2018;1714:237-247). Unaggregated MAVS however looks like tubular structure over mitochondria. In vector control of Fig. 7C, we observed that over 60% cells carry round and concentrated MAVS. We observed that upon co-expression with H7N9 PB1-F2, much MAVS (about 70%) became less round and concentrated but shifted to scattered structure over fissioned mitochondria (Fig. 7C). Those scattered structure gives more pronounced signal compared to non-transfected cells adjacent to the transfected cells (Fig. 7C, panel 13, where some cells give pronounced signal indicative of “scattered MAVS”, whereas low signal is seen in adjacent non-transfected cells give low signal). MAVS was overexpressed in those cells and blocked for aggregation by H7N9 PB1-F2. In addition to confocal assay, SDD-AGE in Fig. 8a provides a quantitative view for how H7N9 PB1-F2 prevents MAVS aggregation to high molecular complex.

Reviewer #2: For the IF confocal microscopy Z stacking is essential to address the distribution of the PB1F2 at mitochondria/mitochondrial aggregates, the same slides could be used to rescan over several vertical planes to allow relevant co-localization distribution

Response: Z-stack analysis has been provided for colocalization of H7N9 PB1-F2 and mitochondrial cluster in Fig. S2.

line 287-289: how do authors explain that H7 PB1-f2 does not precipitate MAVS and has the most pronounced effect on it?

This Must be addressed, is there some third independent factor which promotes aggregation and H7 PB1-F2 sequestrate it??? Or...

line 314-317: If H7 Pb1f2 does not interact with MAVS, why would it suppress signalosome recruitment?

line 338-344: PB1F2 was shown to be heavily ubiquitinated, H7 PB1f2 also contains the C-terminal lysine cluster shown to be responsible for H5 and H1 PB1f2 expression level <https://doi.org/10.1371/journal.pone.0118477>. If H7PB1f2 does not interact with MAVS, authors should investigate if PB1f2 ubiquitination could contribute to MAVS ubiquitination by TRIM31

Response: We tried really hard both in the last two months and well before that, but were still unable to find a definite answer. We cited more references and discussed it further in lines 420-422 and lines 443-448. We have to leave it to the next phase of our study.

figure1. please comment on molecular size differences between wsn H5 and H5 PB1f2, analyze at least at the sequence level. The potential sites for posttranslational modifications like sumoylation, neddylation etc, those could be responsible for altered migration patterns as well as stability.

Response: We have added a sentence to the legend of Figure 1B: “Slight differences in the actual molecular masses of PB1-F2 proteins from WSN, H5N1 and H7N9 viruses on the 12% polyacrylamide gel might be explained at least partially by the differences in their calculated molecular masses”.

Fig.2b: in figure 1b presented data show no substantial difference between H7 PB1f2 regardless of MG132 presence while here fig2b difference is substantial. Please provide an explanation for results inconsistency and quantification with average expression level based on multiple independent repeats of this experiment.

Response: We have resolved the inconsistency by using a more representative blot. We have also added more explanation to the results (lines 136-138 and lines 142-144).

**Part III – Minor Issues: Editorial and Data Presentation Modifications**
Please use this section for editorial suggestions as well as relatively minor modifications of existing data that would enhance clarity.

Reviewer #1: Figure 1 and 2 should be combined.

Response: They have been combined.

Reviewer #2: it would be beneficial if authors could perform co-staining with mitotracker and PB1f2, as pr8 PB1f2 was shown to efficiently co-localize with mitochondria regardless of plasmid transfection or viral infection

Response: Mitotracker staining has been shown in Fig. 2A.

line 88-89 please provide appropriate reference

Response: We have added references (line 88).

line 149-151: despite PR8 PB1f2 is most studied other variants including HK97 H5N1 PB1f2 were. <https://doi.org/10.1371/journal.pone.0118477>
please please edit accordingly

<https://doi.org/10.1099/jgv.0.001220>

please rephrase accordingly

Response: We have edited and rephrased as requested (lines 157-158).

line 240: please specify if co-expression was performed by co-transfection, or infection and transfection

Response: We have revised to indicate that it was through co-transfection (line 275).

line 287-305:

This section is hard to follow, does this mean that aggregation competent H7 PB1f2 interacts with MAVS before it aggregates, and at the steady-state expression of the MAVS, MAVS eventually aggregates making it resistant to H7PB1f2 interaction. Or am I missing something?
Response: We have cited more references and discussed it further in lines 420-422 and lines 443-448.

figure4.: To increase the relevance of luc-assays, please provide wb-data to demonstrate a relative level of PB1F2 along with a relative expression of RIG-IN, MyD88, MAVS TBK-1 IRF3-5D IFNB in the same sample (used for luciferase measurement).

Response: We have included the control in Fig. S3.

figure 9A, B please include PB1-F2 portion of the wb around 12kDa as it has also Flag-taq
TRAF3 has Flag label in front of it while IKK and TKB1 behind it, please unify

Response: We have modified Fig. 8 as suggested.
